# Determinants of utilization of malaria preventive measures during pregnancy among women aged 15 to 49 years in Kenya: an analysis of the Malaria Indicator Survey 2020

**DOI:** 10.1186/s12936-022-04425-x

**Published:** 2022-12-29

**Authors:** Beatrice Mkubwa, Juliana Kagura, Tobias Chirwa, Latifat Ibisomi, Samson Kinyanjui

**Affiliations:** 1 Department of Epidemiology and Biostatistics, School of Public Health, The University of the Witwatersrand, Johannesburg, South Africa; 2 Centre for Geographic Medicine Research (Coast), KEMRI/Wellcome Trust Research Programme, Kilifi, Kenya; 3 Nigerian Institute of Medical Research, Lagos, Nigeria

**Keywords:** Malaria, Pregnancy, Determinants, Prevention, ITN, IPTpSP, Multinomial logistic regression, Kenya

## Abstract

**Background:**

Malaria is a significant cause of morbidity and mortality. Malaria infection in pregnancy can have severe consequences for the fetus and the mother. To fight against malaria infection in pregnancy, Kenya integrated the issuance of an insecticide-treated net (ITN) and intermittent preventive treatment with sulfadoxine-pyrimethamine (IPTpSP) with antenatal care (ANC) for pregnant women. However, the uptake of the ITN and IPTpSP is still low. Individual, social, or structural factors may influence the low uptake. It is, therefore, important to identify the determinants associated with the uptake of ITN and IPTpSP during pregnancy in Kenya.

**Methods:**

Data were from the 2020 Kenya Malaria Indicator Survey (MIS). A total of 1779 women between the ages of 15 to 49 years who had a history of either being pregnant or having given birth within 5 years before the MIS survey were included. Survey-adjusted multinomial logistic regression was used in the analysis.

**Results:**

During pregnancy, ITN use was more than half (54.9%). The use of at least one dose of IPTpSP was 43.5%, three or more doses of IPTpSP was 27.2%, and only 28.2% of the participants used both ITN and IPTpSP during pregnancy. The significant determinants of combined use of ITN and IPTpSP during pregnancy were maternal age (RR 3.57, CI 1.80–7.08; p=<0.001), maternal education (RRR 2.84, CI 1.33–6.06; p=0.007), wealth index (RR 2.14, CI 1.19–3.84; p=0.011) and living in the different malaria epidemiological zones: lake endemic (RRR 10.57 CI 5.65–19.76; p=<0.001), coastal endemic area (RRR 4.86 CI 1.86–12.67; p=0.001), seasonal (RRR 0.21 CI 0.10–0.39; p=<0.001) and low risk (RRR 0.07, CI 0.03–0.17; p=<0.001).

**Conclusion:**

The uptake of malaria preventive measures is still below 80% for both ITN and IPTpSP during pregnancy in Kenya. The significant results on determinants of the use of ITN and IPTpSP could be considered in implementing malaria prevention programmes during pregnancy. For example, sensitizing the community on the importance of antenatal care visits will provide a platform to teach the importance of malaria prevention in pregnancy. Moreover, the pregnant mothers receive an ITN and IPTpSP during the ANC visit.

## Background

Malaria is one of the most common parasitic infectious diseases globally [1, 2]. Approximately 228 million people globally get malaria infection annually [3]. Twenty-five out of the twenty-nine countries that account for 95% of the malaria cases globally are in the World Health Organization (WHO) sub-Saharan African (SSA) countries [2]. Pregnant women, children below 5 years, and infants are the most vulnerable population to contracting malaria [3]. A meta-analysis done in 2015 reported the prevalence of Pregnancy Associated Malaria (PAM) in SSA to be between 10 and 20% [4]. Kenya is among the SSA countries reporting high malaria incidence [5]. More than 30% of inpatient disease diagnoses in the country are related to malaria [6]. In 2019, the Kenya Ministry of Health (MOH) estimated PAM prevalence to be 6.3% among women attending their first Antenatal Care Clinic (ANC) visit [7].

The increased susceptibility to malaria among pregnant women is attributed to the physiological and hormonal changes during pregnancy, causing maternal immunity suppression [8, 9]. If malaria infection in pregnancy is left untreated, it can result in severe consequences, such as fetal intrauterine growth retardation, premature births, low birth weight, and severe maternal anaemia [5, 6]. To prevent malaria during pregnancy, the WHO recommends the use of intermittent preventive treatment with a dose of sulfadoxine-pyrimethamine (IPTpSP) and sleeping under an insecticide-treated net (ITN) [10]. IPTpSP is first given early in the second trimester of pregnancy, at 13 weeks gestation and above, and every 4 weeks in the subsequent ANC clinic visits until the mother delivers [11, 12]. The use of ITN received more attention in the 2000s to prevent malaria [13]. In most sub-Saharan African countries, ITN is given for free through community mass distribution campaigns, at healthcare centres during ANC visits, and through the Expanded Programme of Childhood Immunization [14, 15].

In 1998, Kenya adopted ITN and IPTpSP use for pregnant women. It integrated the two preventive measures with ANC for all pregnant women in public hospitals [16]. Kenya targets coverage of 80% for the use of the ITN and IPTpSP among pregnant women [17]. The Kenya Health Demographic Surveillance (KDHS) carried out between the years 2008 and 2009 show that 49.0% of pregnant women slept under an ITN, and only 33.3% used IPTpSP. In 2015, ITN and IPTpSP use was 57.8% and 50.0%, respectively [18]. From the statistics above, the country still lags behind the 80% coverage target of ITN and IPTpSP set in the Kenya Malaria Strategy 2009–2018 report [17]. Although ITN and IPTpSP are scientifically proven to be efficacious, the use of these preventive measures is still low [18, 19]. The uptake of ITN and IPTpSP may be influenced by individual, social, or structural factors [15, 20]. Therefore, determining these factors may highlight critical gaps in implementing malaria prevention programmes to improve the use of ITN and IPTpSP during pregnancy [21, 22]. This study aimed to examine the use of ITN and IPTpSP and identify the determinants of malaria preventive measures utilization during pregnancy among women aged between 15 and 49 years in Kenya.

## Methods

### Study design and data source

This cross-sectional study utilized the 2020 Kenya Malaria Indicator Survey (MIS) data. Data were collected from 9th November 2020 to 23rd December 2020. Details of the survey are described in the Kenya MIS 2020 report [23]. The MIS survey included a nationally representative sample. Sampling was conducted in two stages: stratified cluster sampling and systematic sampling. The stratified cluster sampling was used to identify 301 clusters, 167 from rural and 134 from urban areas. In the second step, systematic sampling was used to select 30 households from the 301 clusters. Only selected households were interviewed, and replacement of non-responding households was not allowed. A total of 7952 households were successfully interviewed; 6771 eligible women aged between 15 and 49 years selected from the households were consented and interviewed, with a response rate of 97%.

The data collection was done by the Kenya Ministry of Health, the Division of National Malaria Control Programme, by trained fieldworkers using validated MIS questionnaires. The survey collected basic demographic information, birth history, the use of antenatal care services, the use of malaria preventive interventions, and other key malaria indicators. The data sets of the 2020 Kenya MIS were downloaded from the USAID Demographic Health Surveillance Program website in the STATA compatible format [23].

### Study population

**Fig. 1:**
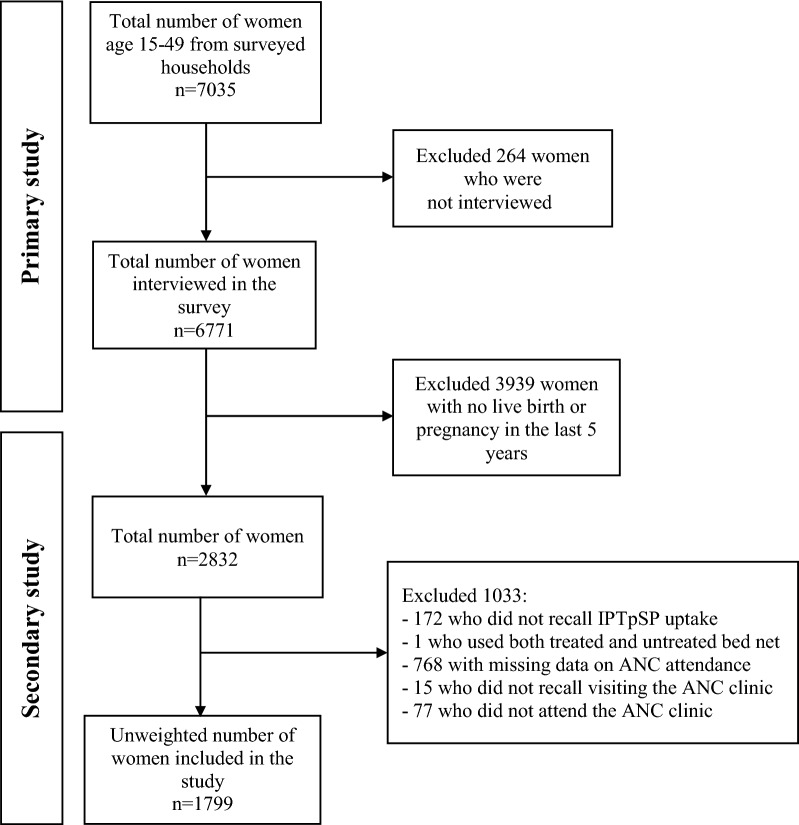
Flowchat diagram showing the selection of study participants included in the primary and this study

A total of 3939 women aged between 15 and 49 years with pregnancy or birth history 5 years before the MIS survey were included in the analysis. The 172 women who did not recall IPTpSP uptake, one who used both treated and untreated bed net, 768 with missing data on ANC attendance, and 15 who did not remember visiting the ANC clinic were excluded. To restrict the data analysis to women who attended ANC, the 77 participants who did not attend the ANC clinic during pregnancy were excluded. After data cleaning, 1799 women (unweighted sample) were included in this study. The inclusion and exclusion criteria used to determine the eligible populations are outlined in Fig. 1.

### Data management

Data extraction and cleaning were done using STATA Standard Edition (SE) version 16. The variables of interest were extracted from the individual recode. The individual recode is the data set for women aged between 15 and 49 years. Based on the literature review, the variables that could influence the uptake of malaria preventive measures during pregnancy were identified from the dataset. Participants with missing data for the outcomes variables of interest were excluded from the study. After data cleaning, the study sample weight was calculated by dividing the household sample weight by 1,000,000 to correct for unequal probabilities of selection that might have occurred during sampling and compensate for survey non-response in the primary survey [24].

### Statistical analysis

Data were analysed using STATA SE version 16. The primary outcomes of interest were ITN uptake, IPTpSP uptake, and combined uptake of ITN and IPTpSP. Participants were asked whether they slept under a mosquito net the night before the survey. ITN use was coded as follows: ‘no bed-net’ and ‘untreated bed-net’, were merged to denote ‘no ITN uptake’ and ‘only a treated net’ to denote ITN uptake. IPTpSP uptake was categorized into a binary variable based on the response to the survey question if the participant took SP/Fansidar during pregnancy to prevent them from getting malaria (no=0 and yes=1) [25]. The variable combined use of ITN and IPTpSP was generated by combining the ‘no’ categories for ITN and IPTpSP use and the ‘yes’ categories of the ITN and IPTpSP use [26]. The explanatory variables included in the analysis were age, parity, residence, educational level, socioeconomic status, religion, exposure to malaria messages, sex of the head of household, and malaria epidemiological zones the participant resides.

Before starting the analysis, the data was survey set using the primary sampling unit, sampling weight, and stratification variables. The prefix’ **svy:’** was used before each command throughout the analysis to account for the stratified cluster sampling used in the primary study.

Survey-adjusted frequencies, proportions, and percentages were used to describe the use of ITN and IPTpSP Pearson’s Chi-Squared test of independence was used to assess the statistical significance and the distribution frequencies of the outcome variables with the independent variables at a 0.05 alpha level. After that, the survey-adjusted multinomial logistic regression was carried out to identify the determinants of utilization of ITN, IPTpSP, and combined use of ITN and IPTpSP during pregnancy.

### Ethical considerations and data privacy

The Kenyatta National Hospital/University of Nairobi Scientific and Ethics Review Committee and the institutional review board at ICF approved the Kenya MIS protocol. In the survey, written informed consent was sought from all participants before administering the questionnaire. Permission to use the 2020 Kenya MIS data for this study was requested from the Measure Demographic and Health Surveys database gatekeepers. The participants' identifiable information was anonymized throughout the survey, and confidentiality was maintained.

## Results

### Bivariate analysis of determinants of uptake of ITN and IPTpSP during pregnancy

Out of the weighted sample of 1671 participants, 54.9% slept under an ITN the night before the survey. The uptake of at least one dose of IPTpSP was 43.5%; at the same time, only 27.2% took up to three or more doses of IPTpSP. Only 28.2% slept under an ITN and took at least one dose of IPTpSP during pregnancy.

Table 1 shows the association between participants’ characteristics and the use of ITN. Use of ITN was higher among women living in the highland epidemic, lake endemic, and coastal endemic zone (more than 65%) than among those living in the seasonal and low-risk malaria zones (37.4% and 39.8%, respectively). Women exposed to malaria messages were more likely to sleep under ITN when pregnant than women who had not seen malaria messages. Furthermore, the use of ITN use the night before the survey among women with a history of live birth or pregnancy in the past 5 years was above 50% among educated women (primary, secondary, or tertiary) compared to women with no education (26.5%).

**Table 1:** Bivariate analysis between participants' characteristics and the uptake of ITN and IPTpSP—Kenya MIS 2020

Explanatory variables	IPTpSP Uptake				ITN Uptake			
	*One or more doses of IPTpSP n=736 n (%)*	*No IPTpSP n=991 n (%)*	*Chi- statisti *^*2*^	*P-value*	*ITN use n = 932 n (%)*	*No ITN use n = 795 n (%)*	*Chi statistic*^*2*^	*P-value*
Maternal age (years)								
15–24	233 (41.2)	333 (58.8)	4.24	0.475	303 (53.5)	263 (46.5)	3.54	0.602
25–34	365 (43.4)	475 (56.6)			456 (54.2)	384 (45.8)		
35–49	128 (48.5)	136 (51.5)			158 (59.9)	106 (40.1)		
Residence								
Urban	240 (39.7)	365 (60.3)	4.43	0.365	320 (52.9)	285 (47.1)	1.63	0.561
Rural	486 (45.6)	580 (54.4)			597 (56.0)	469 (44.0)		
Maternal education								
No education	59 (41.4)	83 (58.6)			38 (26.5)	104 (73.5)		
Primary education	351 (53.0)	311 (47.0)	51.06	**0.002**^a^	373 (55.3)	289 (43.7)	57.25	**0.008**^a^
Secondary education	244 (39.0)	382 (61.0)			375 (59.9)	251 (40.1)		
Tertiary education	73 (30.2)	169 (69.8)			132 (54.6)	109 (45.4)		
Religion								
No religion & other	15 (32.6)	31 (67.4)			20 (43.6)	26 (56.4)		
Roman catholic	110 (39.2)	171 (60.8)	15.89	0.056	162 (57.9)	118 (42.1)	11.22	0.334
Protestant/other christian	537 (43.5)	697 (56.5)			687 (55.7])	546 (44.3)		
Muslim	65 (58.7)	46 (41.3)			47 (42.6)	57 (63.3)		
Wealth index								
Poor	337 (52.6)	303 (47.4)	40.01	**0.035**^a^	345 (54.0)	295 (46.0)	6.18	0.590
Middle-class	130 (40.9)	188 (59.1)			159 (50.0)	159 (50.0)		
Rich	259 (36.4)	452 (63.6)			411 (57.8)	300 (42.2)		
Parity								
Para 2 or less	353 (40.6)	518 (59.4)	21.09	**0.020**^a^	497 (57.0)	374 (43.0)	3.76	0.700
Para 3 & 4	222 (42.0)	306 (58.0)			276 (52.63)	252 (47.7)		
Para 5 & 6+	151 (55.5)	121 (44.5)			144 (52.9	128 (47.1)		
Exposure to malaria messages								
No	406 (38.3)	653 (61.7)	33.30	**<0.001**^a^	534 (49.1)	526 (50.9)	25.48	**0.005**^a^
Yes	320 (52.4)	291 (47.6)			383 (62.7)	228 (37.3)		
Number of ANC visits								
One ANC visit	10 (33.0)	21 (67.0)	7.12	0.397	16 (51.7)	15 (48.3)	11.59	0.275
Two ANC visits	46 (40.9)	66 (59.1)			70 (62.0)	43 (38.0)		
Three ANC visits	181 (39.6)	276 (60.4)			223 (48.8)	233 (51.2)		
Four or more ANC visits	490 (45.7)	582 (54.3)			609 (56.8)	463 (43.2)		
Household head sex								
Male	517 (43.0)	685 (57.0)	0.41	0.749	680 (56.5)	523 (43.5)	5.20	0.319
Female	209 (44.7)	259 (55.3)			236 (50.6)	231 (49.4)		
Malaria epidemiological zones								
Highland epidemic-prone	111 (36.7)	191 (63.3)	623.57	**<0.001**^**a**^	213 (70.8)	87.9 (29.2)	184.79	**<0.001**^a^
Lake endemic	341 (86.8)	52 (13.2)			285 (72.5)	107 (27.5)		
Coastal endemic	106 (80.8)	25 (19.2)			87 (66.2)	44 (33.8)		
Seasonal	61 (28.2)	156 (71.8)			81 (37.4)	135 (62.6)		
Low risk	107 (17.1)	521 (82.9)			250 (39.8)	387 (60.2)		

*P-values are from chi-squared tests. IPTpSP is Intermittent preventive treatment with Sulfadoxine Pyrimethamine in pregnancy*

*ITN* insecticide treated net*, ANC* antenatal care*, RRR* Relative Risk Rate

^*a*^p-value less than 0.05 is in bold

As shown in Table 1, maternal education, parity, exposure to malaria messages, wealth, and the malaria epidemiological zone the participants reside in were significantly associated with the uptake of the IPTpSP during pregnancy. More than half of the women with primary school education took at least one dose of IPTpSP; however, the uptake was 39.0% and 30.2% with secondary and tertiary education, respectively. The rich were less likely to use IPTpSP (36.4%) than the poor (52.6%).

### Multinomial analysis of determinants of uptake of ITN and IPTpSP during pregnancy

The multinomial logistic regression results are shown in Table 2. The results indicate that participants whose highest education was secondary and identified as rich were more likely to use ITN the night before the survey (RRR 4.64 CI 1.17–18.45; p=0.030 and RRR 2.50 CI 1.42–4.42; p=0.002, respectively). Additionally, women living in seasonal and low-risk malaria zones were 72% and 86%, respectively, less likely to use ITN only (RRR 0.28; CI 0.12–0.64; p=0.002 and RRR 0.14; CI 0.06–0.30; p=<0.001).

**Table 2:** Determinants of use of malaria preventive measures during pregnancy in Kenya – MIS 2020.

Explanatory variables	ITN use versus no use			IPTpSP uptake versus no use			Combined uptake—IPTpSP & ITN versus no use		
	*Relative risk ratio (RRR)*	*95% confidence interval (CI)*	*P>[z]*	*Relative risk ratio (RRR)*	*95% confidence interval (CI)*	*P>[z]*	*Relative risk ratio (RRR)*	*95% confidence interval (CI)*	*P>[z]*
Maternal age (years)									
15–24	Ref			Ref			Ref		
25–34	1.24	0.58–2.66	0.576	1.47	0.82–2.64	0.196	2.00	1.09–3.67	**0.026**^**a**^
35–49	2.31	0.99–5.41	0.053	1.88	0.85–4.16	0.117	3.57	1.80–7.08	**<0.001**^**a**^
Residence									
Urban	Ref			Ref			Ref		
Rural	1.53	0.91–2.57	0.110	0.81	0.41–1.60	0.542	1.23	0.63–2.40	0.548
Maternal education									
No education	Ref			Ref.			Ref		
Primary	3.25	0.93–11.41	0.065	0.98	0.35–2.71	0.961	2.84	1.33–6.06	**0.007**^**a**^
Secondary	4.64	1.17–18.45	**0.030**^**a**^	0.79	0.24–2.58	0.693	2.87	1.16–7.15	**0.023**^**a**^
Tertiary	3.06	0.65–14.32	0.155	0.27	0.07–0.96	**0.044**^**a**^	0.88	0.27–2.82	0.827
Religion									
No religion & other	Ref			Ref			Ref		
Roman Catholic	0.78	0.27–2.25	0.640	0.51	0.12–2.07	0.342	1.95	0.52–7.31	0.318
Protestant or other christian	0.53	026–1.11	0.092	0.51	0.18–1.39	0.186	1.07	0.39–2.92	0.890
Muslim	0.60	0.23–1.55	0.290	0.99	0.30–3.28	0.987	2.03	0.59–6.99	0.262
Wealth index									
Poor	Ref			Ref			Ref		
Middle-class	0.73	0.34–1.61	0.439	0.60	0.29–1.25	0.173	0.74	0.34–1.62	0.446
Rich	2.50	1.42–4.42	**0.002**^**a**^	1.57	0.85–2.91	0.147	2.14	1.19–3.84	**0.011**^**a**^
Parity									
Para 2 or less	Ref			Ref			Ref		
Para 3 & 4	0.72	0.20–2.52	0.605	0.69	0.35–1.35	0.279	0.58	0.28–1.21	0.146
Para 5 & 6 above	0.43	0.11 -1.68	0.226	0.50	0.25–0.98	**0.044**^**a**^	0.61	0.31–1.21	0.155
Exposure to malaria messages									
No	Ref			Ref			Ref		
Yes	0.99	0.59–1.65	0.970	1.30	0.74–2.27	0.370	1.46	0.87–2.47	0.154
Number of ANC visits									
One ANC visit	Ref			Ref			Ref		
Two ANC visits	2.90	0.49–17.00	0.239	4.30	1.07–17.24	**0.040**^**a**^	2.46	0.52–11.68	0.256
Three ANC visits	1.01	0.24–4.30	0.987	2.85	0.77–10.51	0.115	1.73	0.44–6.91	0.433
4 or more ANC visits	0.85	0.22–3.22	0.810	1.89	0.54–6.63	0.321	1.84	0.49–6.86	0.363
Household head sex									
Male	Ref			Ref			Ref		
Female	0.82	0.34–1.94	0.649	1.17	0.65–2.13	0.599	0.91	0.45–1.84	0.792
Malaria epidemiological zones									
Highland epidemic-prone	Ref			Ref			Ref		
Lake endemic	0.79	0.37–1.71	0.865	8.99	4.92–16.43	**<0.001**^**a**^	10.57	5.65–19.76	**<0.001**^**a**^
Coastal endemic	0.58	0.21–1.61	0.225	4.14	1.47–11.67	**0.007**^**a**^	4.86	1.86–12.67	**0.001**^**a**^
Seasonal	0.28	0.12–0.64	**0.002**^**a**^	0.38	0.15–0.99	**0.047**^**a**^	0.21	0.10–0.39	**<0.001**^**a**^
Low risk	0.14	0.06–0.30	**<0.001**^**a**^	0.20	0.09–0.47	**<0.001**^**a**^	0.07	0.03–0.17	**<0.001**^**a**^

*Ref. is the Reference Category*

*IPTpSP* Intermittent preventive treatment with Sulfadoxine Pyrimethamine in pregnancy, *ITN* insecticide treated net, *ANC* antenatal care, *RRR* Relative Risk Rate

^*a*^for significant p-values at 5% alpha level is in bold

IPTpSP use among participants living in lake endemic and coastal endemic regions was significantly higher than those living in the low-risk malaria epidemiological areas, which were 80% (RRR 0.20; CI 0.09–0.47; p=<0.001) less likely to use IPTpSP while pregnant. Participants with tertiary level education were less likely to use IPTpSP during pregnancy versus not using any malaria preventive measure (RRR 0.27 CI 0.07–0.96; p=0.044). Advanced maternal age (35 to 49 years) predicts higher uptake of both IPTpSP and ITN when pregnant (RRR 3.57; CI 1.80–7.08; p=<0.001). Participants with primary and secondary education were more likely to use ITN and IPTpSP than those without formal education. Participants who lived in coastal and lake endemic malaria zones were more likely to use both ITN and IPTpSP when pregnant than women living in the seasonal and low-risk malaria zones than women living in the highland epidemic region. Furthermore, participants whose wealth index was rich were more likely to use both ITN and IPTpSP than the poor (RRR 2.14; CI 1.19–3.84; p=0.011).

## Discussion

This study examined the extent of use and the determinants of ITN and IPTpSP uptake among pregnant women in Kenya using data from the 2020 MIS. The findings show that the uptake of ITN (54.9%) was higher than three or more doses of IPTpSP (27.2%), and only 28.2% of the study population used both ITN and at least one dose of IPTpSP. The uptake of ITN recorded in this study is slightly higher than the 50.6% uptake reported in the 2014 Kenya Demographic Health Survey (KDHS); however, these numbers are below the 80% target recommended by the WHO guidelines [18]. In KDHS reports, the IPTpSP uptake was 6.9% in 2008/2009, 10.3% in 2014, and 22.9% in 2015. The study findings show that around a quarter of the women analysed took three or more doses of IPTpSP. Compared to some SSA countries, the uptake of IPTpSP in Kenya is slightly lower. For example, in the Uganda 2018-2019 MIS, 41.0 % of the participants took three or more doses, while in Sierra Leone, the uptake was 35.7% [27, 28]. Despite reports that combining ITN and IPTpSP during pregnancy significantly reduces malaria-related complications in pregnancy than using either ITN alone or IPTpSP, the uptake of using both interventions remains very low in Kenya [18, 29, 30].

Determining the factors influencing the use of malaria preventive measures during pregnancy might inform early, frequent and appropriate delivery of ITN and IPTpSP, which is essential in the fight against malaria infection [31]. This study revealed that the use of ITN and IPTpSP were significantly associated with maternal education, wealth index, maternal age, and living in Kenya’s endemic, seasonal or low-risk malaria zones. ITN use was significantly high among participants whose highest level of education was secondary. However, it was noted that women with a tertiary education were 73% less likely to use IPTpSP while pregnant. An individual’s education level has been linked to the uptake of health interventions [32]. Education is not the sole predictor; other factors, such as income, interact in many important ways with education and influence health interventions’ uptake [32, 33]. In this study, it was found that women with five children or more were 50% less likely to use IPTpSP when pregnant, contrary to findings from a study done in Tanzania [34].

Results suggest that women living in Kenya's low-risk and seasonal-risk malaria epidemiological regions are less likely to use malaria preventive measures than those in the higher epidemic zone. In seasonal malaria transmission areas in Kenya, the risk of getting malaria is less than 5%. In the low-risk malaria areas, it is 0.1% [6]. Therefore, people living in low-risk malaria regions might not perceive malaria infection as life-threatening and might not sleep under an ITN or take IPTpSP while pregnant. To note is that the Kenya National Malaria Control Strategy omits IPTpSP in these lower-risk zones. Hence, women in these areas are unlikely to have access to the intervention. Consequently, the uptake of malaria preventive measures is lower in areas with less malaria risk [6]. Other studies have also found similar evidence for lower uptake of malaria preventive measures during pregnancy in the areas of lower risk of malaria [35].

Evidence of a possible link between maternal age and the uptake of malaria preventive measures in pregnancy has been reported in previous studies. It is argued that older women might be more knowledgeable about the risk of malaria and, therefore, display higher usage of ITN and IPTpSP during pregnancy [36]. Similarly, in this study, the relative risk ratio of maternal age was a significant determinant of the combined uptake of ITN and IPTpSP during pregnancy. The findings also showed that the participants categorized as rich were likely to sleep under ITN and use both malaria preventive measures. Previous studies have reported that women classified as poor may have little or no access to the antenatal care clinic due to a lack of transport costs and payments at the clinic [37]. Therefore, access to the freely administered IPTpSP and a free issue of ITN during pregnancy at the ANC clinic might not be possible [37, 38].

The study found that women who had two ANC clinic visits were likely to use IPTpSP but not ITN or combined use of ITN and IPTpSP compared to those who visited the clinic once. Similar findings have been reported where IPTpSP use was associated with ANC visits but not ITN use during pregnancy [36]. In Uganda, a study that looked at the determinants of utilization of ITN and IPTpSP reported that women who attended the ANC clinic were more likely to use malaria preventive measures [38]. On the contrary, a study that looked at the uptake of ITN and IPT in Gabon did not find any association between ANC clinic attendance and the uptake of malaria preventive measures during pregnancy [39]. Attending the ANC clinic offers an opportunity for pregnant women to be educated about the dangers of malaria during pregnancy and therefore make informed health decisions about the uptake of the preventive measures [20]. Nevertheless, multiple ANC clinic visits do not guarantee high uptake of IPTpSP during pregnancy [40]. This could be due to missed opportunities to administer IPT due to regular stock-outs at the ANC clinics or late/infrequent visits to the clinic [30].

The findings show that living in rural or urban areas was not a determinant of IPTpSP, ITN, or combined use of ITN and IPTpSP during pregnancy. This is in contrast to a study done in Ethiopia that found an association between the type of residence and ITN use among pregnant women [19]. The gender of the household head was also not a significant determinant of the uptake of malaria preventive measures. However, it's been argued that in some communities in Kenya, women might not have the autonomy to make decisions, even regarding their health, without consulting the head of the household [26]. Last, religion was not found to be a significant determinant of ITN and IPTpSP. On the other hand, studies on factors affecting ITN and IPTpSP utilization in Kenya and Ghana found a significant association between religion and ITN utilization but not IPTpSP uptake [5, 6]. Future research may explore further examination of the impact of religion on the use of malaria preventive measures in Kenya.

One of the strengths of this study is that the Malaria Indicator Survey is a standardized malaria surveillance tool, and the participants are representative of the national sample. However, this study had several limitations. First, this is a cross-sectional study; therefore, the study could not demonstrate a causal relationship between the outcome and the explanatory variables. Secondly, the data collected during the Kenya Malaria Indicator Survey was self-reported and therefore was subject to recall bias. Thirdly, this research relies on the quantitative approach only. Consequently, it was not possible to qualitatively understand the factors determining malaria preventive interventions during pregnancy, such as behavioural aspects and motivation. This is a secondary data analysis study; therefore, the number of available variables in the Malaria Indicator Survey questionnaire was limited. Hence residual confounding could not be ruled out, for example, the availability of ITN and IPTpSP drugs in the ANC clinic and the participants’ marital status and occupation.

## Conclusion

To conclude, ITN and IPTpSP use is still below the 80% uptake recommended by the WHO guidelines. Understanding the determinants of ITN and IPTpSP use during pregnancy before designing strategies to improve the uptake of these measures. For example, sensitization on the importance of ANC visits during pregnancy is significant in the fight against malaria pregnancy. The ANC visits provide an opportunity for pregnant women to be educated in ITN and IPTpSP and are given ITN and IPTpSP during the visit. The MOH and health stakeholders need to scale up national campaigns and create community awareness of malaria preventive measures during pregnancy to increase utilization. Conducting studies to explore qualitative perspectives and the factors influencing the uptake of malaria preventive measures in pregnancy in Kenya is recommended.

## Data Availability

The data used was collected during the 2020 Kenya Malaria Indicator Survey, which is available from the DHS program database gatekeepers upon request at their website, http://www.dhsprogram.com.

## Abbreviations

ANC: Antenatal care clinic
DHS: Demographic health surveillance
IPTpSP: Intermittent preventive treatment in pregnancy with sulfadoxine pyrimethamine
ITN: Insecticide treated net
MIS: Malaria indicator survey
MLR: Multinomial logistic regression
MOH: Ministry of health
PAM: Pregnancy associated malaria
SSA: Sub-Saharan Africa
USAID: United states agency for international development
WHO: World Health Organization
